# Comparison of Neurodevelopmental Therapy with Standard Therapy for the Treatment of Patients with Spasticity After Stroke

**DOI:** 10.3390/jcm14103450

**Published:** 2025-05-15

**Authors:** Rafał Studnicki, Maciek Krawczyk, Rita Hansdorfer-Korzon, Igor Z. Zubrzycki, Magdalena Wiacek

**Affiliations:** 1 Department of Physiotherapy, Medical University of Gdańsk, 7 Dębinki Street, 80-211 Gdańsk, Poland; rita.korzon@gumed.edu.pl; 2 Faculty of Rehabilitation, University of Physical Education, 00-968 Warsaw, Poland; maciej.krawczyk@awf.edu.pl; 3 IInd Department of Neurology, Institute of Psychiatry and Neurology, 02-957 Warsaw, Poland; 4 Department of Health and Medical Sciences, Radom University, Chrobrego 27, 26-600 Radom, Poland; i.zubrzycki@urad.edu.pl

**Keywords:** stroke, neurodevelopmental therapy, rehabilitation, MAS

## Abstract

**Background/Objectives:** The objective of this study was to expose the ability of neurophysiotherapeutic management to reduce spasticity through a modified Ashworth Scale. **Methods**: The sample, consisting of 102 subjects divided into control and study groups, was selected from an initial pool of N = 1007 patients diagnosed with stroke by a neurosurgeon that was later confirmed with imaging (MRI, CT). The study scheme included statistical differentiation between the study and control groups before and after applying specific rehabilitation programs and statistical differentiation within these groups before and after their rehabilitation procedures. **Results**: The results of this study revealed statistically significant improvements in reducing spasticity, as assessed by the Ashworth scale, within the group that participated in the neurorehabilitation program. It also confirmed that using neurophysiological methods is a highly effective approach to managing spasticity in post-stroke patients. **Conclusions**: Using neurophysiological methods in the standard physiotherapy treatment of spasticity is very effective for managing post-stroke spasticity.

## 1. Introduction

In healthy individuals, the motor cortex activates the agonist and antagonist muscles during any activity [1]. In post-stroke patients who develop spasticity, the upper motor neurons are damaged, causing upper motor neuron syndrome (UMNS), which results in a loss of reciprocal inhibition. This phenomenon makes patients with spasticity unable to produce force and movement, significantly reducing their independence and affecting their activities of daily living [2]. Restoring daily autonomy is an essential goal of patient rehabilitation. During rehabilitation, complications can occur, including comorbidities and spasticity-related complications, such as muscle contracture [3]. Changes that influence the impediment of volitional movement are a) the structure of muscle and tendon fibers and b) the morphological structure of intracellular and extracellular components [1]. The complexity of spasticity requires the application of a centrally acting muscle relaxant such as intrathecal or baclofen, a neuromuscular blocking agent (botulinum toxin injections), or a neurolytic agent used for nerve blocking (alcohol injection); surgical intervention; and rehabilitation [4]. Rehabilitation programs aim to reduce spasticity and improve the range of motion of the joint, mobility, comfort, and cardiovascular endurance. The fundamental element included in all spasticity treatments is physical therapy, which is founded on the active participation of the patient and often supported by the patient’s family or caregiver. Its primary role is to help increase independence and rebuild the patient’s ability to perform daily activities unassisted. Verification of the outcome of physical therapy is achieved through periodic progress controls. A prerequisite for such controls is the repeatability of the conditions under which the patient is examined. Since factors such as fatigue, time of day, and environmental conditions can affect the level of spasticity a person experiences, subsequent control examinations must be performed under conditions similar to the initial examination [3,4].

During the second half of the twentieth century, a variety of methods, such as NDT-Bobath (Neurodevelopment Treatment) [5] and PNF (proprioceptive neuromuscular facilitation) [6], were developed and are now employed in the treatment of patients with spasticity.

Drawing upon the principle of motor center coordination between the upper and lower extremities, the originators of proprioceptive neuromuscular facilitation (PNF) developed a structured set of exercises intended to elicit reflex locomotion mechanisms [7]. The NDT-Bobath approach is also applicable to spasticity in patients with motor dysfunction. It is based on actively encouraging patients to develop motor control using key points and reflex inhibition patterns [7]. Therefore, instead of passive activity, the patient performing the active exercise engages in the tensing of their antagonist muscles. Thus, cooperation between the agonist and antagonist muscles is facilitated [8]. The basic principles behind the treatment of patients with spastic muscle tension focus on avoiding harmful stimuli and achieving a specific range of movement [9,10]. Physiotherapeutic interventions, such as kinesiotherapy, can reduce spasticity, allowing the return of motor function [11]. Specialized approaches focus on normalizing muscle tone and motor pattern learning [12]. Therapeutic approaches use the phenomenon of the compensatory (post-damage) plasticity of the nervous system, which refers to the ability of the nervous system to self-repair through the formation of new connections and the re-recruitment of nerve cells. Due to plasticity, another area of the brain can adopt activities that were specific to a different brain area, allowing a particular activity to return [12]. Thus, physiotherapists must consider whether it is enough to relax muscles with pathologically increased tension or whether patients should be taught the de novo contraction of specific muscles [11]. The neurodevelopmental approach requires many hours of therapeutic care. During this process, a physical therapist selects particular elements of the neurodevelopmental method that could be relevant for a patient and uses them during the therapeutic session, regulating muscle tone and restoring functional movement through the principles of motor learning. Therapy time is divided into working on posture positions, patient hygiene, and gait training [13]. However, the effectiveness of proprioceptive neuromuscular facilitation (PNF) [6,13] or the NDT-Bobath method [14,15] in the rehabilitation of patients with stroke has only been documented in a few studies [8,16,17].

Due to the small number of reports dedicated to the clinical management of stroke patients with spasticity, and particularly to the assessment of spasticity using the modified Ashworth scale (MAS), this study evaluated the impact of PNF and NDT-Bobath on rehabilitation progress. We hypothesized that neurophysiotherapeutic management should visibly reduce spasticity, as assessed using the modified Ashworth Scale (MAS). The MAS was selected over the original Ashworth Scale due to its enhanced sensitivity to subtle changes in muscle tone, especially in patients with mild to moderate spasticity. The inclusion of an intermediate grade (1+) allows for the more precise assessment and monitoring of spasticity progression and treatment effects in clinical and research settings.

## 2. Materials and Methods

### 2.1. Ethical Standards

This study received ethical approval from the Independent Bioethics Committee for Scientific Research at the Medical University of Gdańsk (Resolution No. NKEBN/76-271/2022, 22 April 2022). All participants were provided with detailed information regarding the study objectives and procedures. Prior to participation, written informed consent was obtained from each participant or their legal guardian. This study was carried out in line with the ethical principles outlined in the Declaration of Helsinki.

### 2.2. Participants

Patients (N = 1007) diagnosed with stroke by a neurosurgeon that was confirmed via imaging (MRI, CT) were eligible for our initial analysis. The pre-qualification stage of this study took place over the telephone, during which an interview was conducted to assess the patients’ level of spasticity. The questions asked included statements about the difficulty of raising the arm above the head, grasping an object independently, and whether there was difficulty walking. Pre-qualified people were visited by a physiotherapist at their home and interviewed, with an assessment of their spasticity made according to the Ashworth scale [1]. The modified Ashworth Scale, a six-point numerical scale, assesses increased muscle tone (spasticity).

Inclusion criteria: first-ever ischemic stroke resulting in hemiparesis on one side of the body, spasticity of at least two on the modified Ashworth Scale (MAS) in at least one muscle group, NIHSS score 15, Rankin scale 3, Barthel scale 55, 18 years of age or older, and medical rehabilitation in a rehabilitation department.

Exclusion criteria: a diagnosis of transient ischemic attack (TIA); a confirmed second or subsequent stroke; the presence of comorbid conditions that could potentially influence the development of spasticity, such as malignancy, myocardial infarction, or conditions requiring analgesia; lack of consent from the patient’s physician or family; and an absence of medical rehabilitation conducted within a rehabilitation facility. Patients were excluded from the study for the following reasons: absence of spasticity, refusal to be contacted by a researcher, and refusal to participate in the study. Initially, 396 patients were assessed for eligibility. Among these, exclusions were made due to poor general health status (N = 55), an inability to establish verbal communication (N = 125), a history of a subsequent stroke (N = 58), and an unwillingness to cooperate (N = 56). Following these exclusions, the final study sample included 102 patients diagnosed with post-stroke spasticity. Participants were allocated to two different groups based on the type of therapeutic intervention they received.

#### Independent Variables

Participants were allocated to two study groups according to the type of post-stroke rehabilitation they received.

Study Group (SG, “neuro”): This group consisted of 51 patients (21 males and 30 females) with a mean age equal to 69.5 and a standard deviation equal to 10.66. Within this group, 7 patients were diagnosed with aphasia. Hospital-based rehabilitation was provided to the SG within six months of each patient’s first stroke. After discharge from the acute neurology stroke unit, these patients awaited transfer to a rehabilitation hospital, where they subsequently received inpatient rehabilitation. The rehabilitation program followed standard clinical protocols and included neurofunctional training that included elements of proprioceptive neuromuscular facilitation (PNF) and Bobath Neurodevelopmental Therapy (BNT) for both upper and lower limb rehabilitation. Additional therapeutic activities comprised gait training, self-care training, and ergometer cycling. The intervention was delivered over six weeks, with daily sessions lasting 45 min, and included passive and active exercises for the upper and lower extremities.

The rationale behind the use of PNF for reducing spasticity was based on the following observations: PNF techniques encourage the activation of antagonist muscles (e.g., extending muscles to counter spastic flexors), which leads to reciprocal inhibition—a neurologic process that inhibits spastic agonists and helps normalize muscle tone. PNF exercises are based on natural movement patterns, such as diagonals and spirals, which are more reflective of everyday activities and engage multiple joints and muscle groups. This helps improve motor control and reduce the synergy of pathological movement. Moreover, PNF enhances motor relearning, regulates muscle tone, and improves proprioceptive feedback.

Control Group (CG, “normal”): The control group comprised 51 patients (35 males and 16 females) with a mean age of 66.4 and a standard deviation of 11.25, who did not receive hospital-based rehabilitation within the first six months following their stroke. Within this group, 9 patients were diagnosed with aphasia. Upon discharge from the acute neurology stroke unit, these individuals remained at home, where their physical activity was encouraged by family members or caregivers (e.g., spouses and children). This group performed standard exercises daily for six weeks, including passive limb mobilization, gait training, and ergometer use. However, no structured neurophysiological interventions such as PNF or BNT were administered.

### 2.3. Procedures

Initially, evaluators (neurologists) conducted the post-stroke surveys. Six months after their stroke, during admission to the rehabilitation unit, patients’ spasticity status was assessed using the modified Ashworth Scale (MAS). Each assessment was performed individually and by the same person, who had 20 years of experience in neurological physiotherapy. The evaluation was conducted in a quiet room without the presence of other participants.

The methodology used for assessing spasticity in the upper limbs was as follows:

Fingers: The patient was examined in a sitting position. The patient’s shoulder girdle was fully relaxed, their arm and forearm were positioned neutrally, and their fingers were in maximum flexion. The examiner, grasping the patient’s metacarpus and II-V fingers, performed a passive flexion movement of the II-V fingers from maximum flexion to maximum extension.

Thumb: The patient was examined in a sitting position. The patient’s shoulder girdle was completely relaxed; their arm, forearm, wrist, and II-V fingers were positioned neutrally; and their thumb was maximally flexed. The examiner grasped the patient’s metacarpus and thumb and performed a passive thumb extension from maximum flexion to maximum extension.

Wrist: The patient was examined in a sitting position. The patient’s shoulder girdle was fully relaxed, their arm and forearm were positioned neutrally, and their wrist was in maximum flexion. The examiner, grasping the patient’s distal forearm and holding the patient’s hand with their other hand, performed a passive extension movement at the radial–carpal joint to maximum extension.

Supination: the patient was examined in a sitting position. The patient’s shoulder girdle was fully relaxed, their arm was in a neutral position beside the trunk, and their forearm was in maximum recoil. The examiner, grasping the patient’s distal arm and distal forearm from the palmar side, performed a passive inversion movement from maximal supination to maximal supination.

Elbow: The patient was examined in a sitting position. The patient’s shoulder girdle was completely relaxed, and their arm was positioned neutrally beside the body at maximum elbow joint flexion. The examiner, grasping the patient’s midarm and distal forearm from the dorsal side, performed a passive stretching movement at the elbow joint to maximum extension.

Shoulder: The patient was examined in a sitting position. The patient’s shoulder girdle was fully relaxed, and their arm was positioned neutrally beside the torso. The examiner, grasping the distal side of the patient’s arm and forearm, performed a passive abduction movement of the shoulder joint to maximum abduction.

The methodology used for assessing the spasticity in the lower limbs was as follows:

Toes: The patient was examined in a supine position. Their hip joint was in a neutral position in the sagittal and horizontal plane, and their knee joint was straight to the toes of the foot in flexion. The examiner stabilized the patient’s metatarsus distally, grasped toe V, and then passively moved the toes to their maximum extension.

Foot: The patient was examined in a supine position. The hip joint of their impaired limb was placed in a neutral position in the sagittal plane and in natural rotation, and the foot was examined in a position of maximum flexion. The examiner, stabilizing the patient’s lower leg distally and holding their foot with a lumbar grip, performed a passive motion of foot from maximum extension to maximum flexion.

Knee: The patient was examined in a supine position. Their hip joint was placed in a neutral position in the sagittal and horizontal planes and their feet were positioned off the end of the bench. The examiner, stabilizing the patient by their distal thigh and grasping their distal shin, performed a passive straightening movement in the knee joint, moving from maximum flexion to maximum extension.

Hip: The patient was examined in a supine position. Their hip joint was placed in a neutral position (in the frontal plane) and a neutral position in the horizontal plane. The examiner, grasping the patient by the distal part of their thigh and shin on the adductor side, performed a passive lower limb movement to maximal abduction.

#### Statistical Procedures

**Figure 1:**
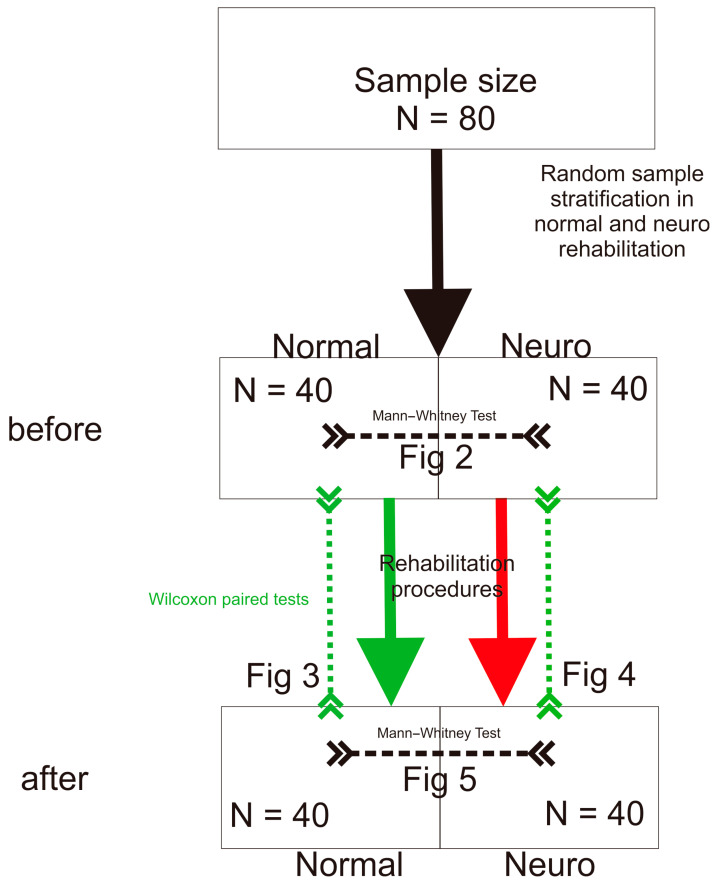
Flowchart of participants (Figure 2, Figure 3, Figure 4 and Figure 5).

All statistical analyses were performed using the R package [18] and the following statistical procedures were used to extract statistical inferences: the Mann–Whitney test [19] and Wilcoxon test [20]. These statistical analyses are shown in Figure 1.

## 3. Results

**Figure 2:**
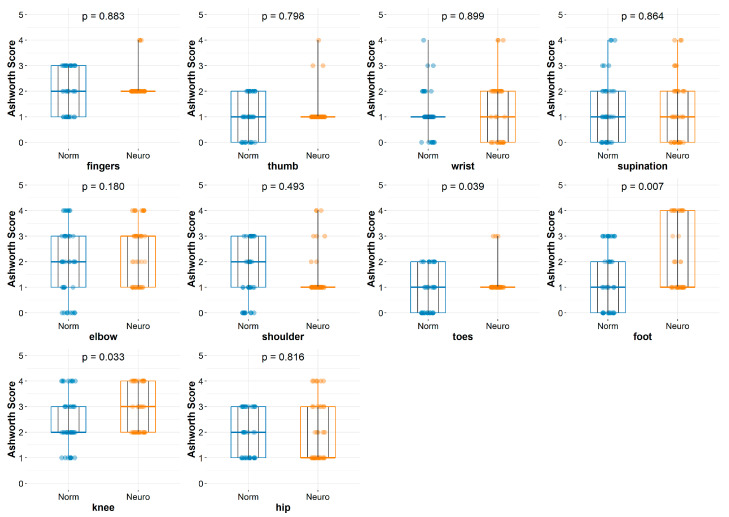
Graphs describing differences between groups before rehabilitation. MAS: modified Ashworth Scale. SG: study group (neuro); CG: control group (normal).

Figure 2 and Figure 5 show the differences between the groups before and after the rehabilitation procedures. Repeated measurements are depicted as broken black lines. For clarity, only 10 specific points are shown on the graph. Thus, Figure 2 presents the differences in muscular spasticity before applying a particular treatment between the groups, which were labeled as “normal” and “neuro” rehabilitation. It reveals a lack of statistically significant differences in all measurements except knee tension (*p* = 0.033), toe tension (*p* = 0.039), and foot tension (*p* = 0.007). In Figure 5, the graphical representation of the differences in muscle spasticity between the groups, identified as “normal” and “neuro” rehabilitation, after applying specific rehabilitation treatments reveals statistically significant differences for all the entities measured, except for wrist and elbow spasticity, with *p* = 0.539 and 0.431, respectively.

**Figure 3:**
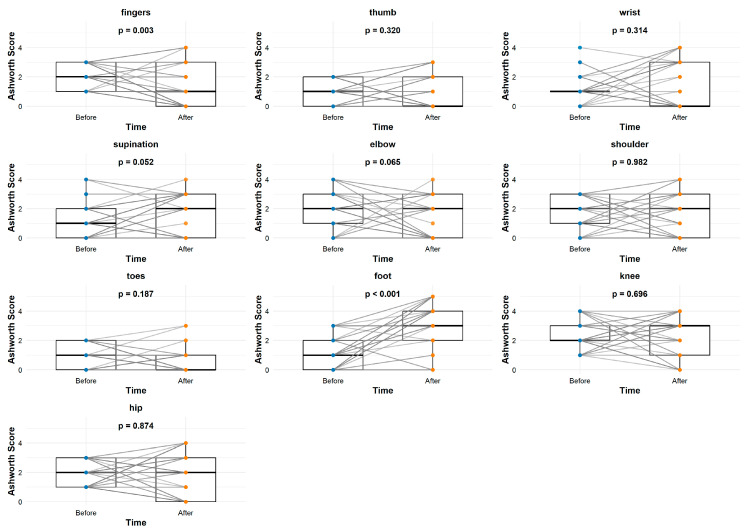
Graphs describing therapeutic differences before and after 6 months. MAS: modified Ashworth scale. CG: control group (normal).

**Figure 4:**
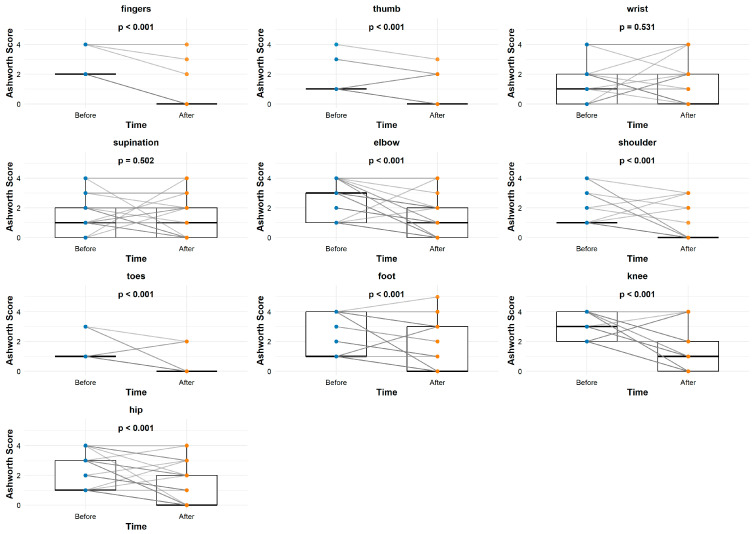
Graphs describing therapeutic differences before and after 6 months. MAS: modified Ashworth scale. SG: study group (neuro).

**Figure 5:**
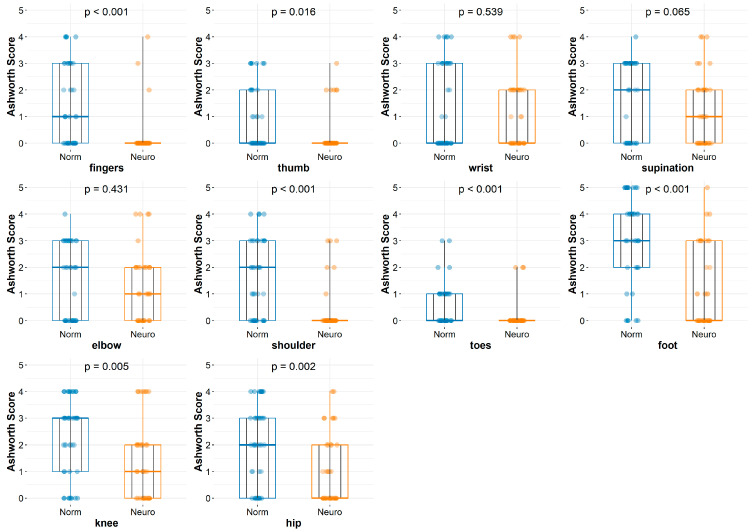
Graphs describing differences between groups after rehabilitation. MAS: modified Ashworth scale. SG: study group (neuro); CG: control group (normal).

Figure 3 shows the changes within the “normal” group after applying the standard rehabilitation treatment. The analysis of Figure 3 reveals the presence of a lack of significant improvement in spasticity across all studied body parts, with the exceptions of the fingers (*p* = 0.003) and foot (*p* < 0.001). Figure 4 shows the differences obtained when using neural rehabilitation procedures. There is a clearly visible improvement across the majority of body parts, with the exceptions of the wrist (*p* = 0.531) and toes (*p* = 0.502).

## 4. Discussion

The present study examines the relationship between exercise type and spasticity and contributes to the broader discourse on the effectiveness of neurodevelopmental exercises in stroke rehabilitation. Hence, the outcome of this study may influence the development of a novel practical neurorehabilitation process. The impact of the methods used to treat spasticity remains a complicated research topic due to the reduced range of motion or spasticity in these patients [21,22]. However, an adjustment in kinesiotherapy has been shown to affect the spasticity of the affected limb and increase its motor and endurance abilities [6]. Furthermore, to make a patient more independent in their daily life [23], therapy should be carried out to reduce spasticity and prevent contractures.

According to previous studies, muscle contracture appears in the second week after a stroke and intensifies within the following two months [24]. This condition can persist during the first year after a stroke. The consequences of these phenomena are rheological changes in the muscles, which may appear beyond the first year after a stroke [25,26]. It is hypothesized that neurofunctional exercises should prevent these detrimental changes [27]. For example, Gomez et al. [2] conducted a meta-analysis of the effects of various stretching exercises and found that this intervention alone was not effective enough to create statistically significant differences that could be observed using the modified Ashworth scale. In 2018, Mahmood et al. [28] published a comprehensive review and meta-analysis that revealed the efficacy of specific rehabilitation procedures in reducing spasticity in the lower extremities for long-term stroke survivors. Another meta-analysis [29] evaluated the effects of dry needling (DN) on muscles in patients after a stroke. However, very moderate evidence indicated that DN positively reduces spasticity in the lower extremities of patients with spasticity.

Furthermore, no effect on motor function was observed. In a study conducted by Brusola et al. [30], the authors found that rehabilitation should prioritize active strategies over passive interventions. The observations made in previous reports were confirmed in this extensive study, showing that even a small amount of weekly patient-centered neurofunctional rehabilitation can lead to reduced spasticity. In addition, our report confirms that well-established rehabilitation is based on evidence-based practice. It also adheres to evidence-based clinical practice recommendations as a guideline for therapeutic management [31]. It also reveals that healthcare professionals need more education about evidence-based stroke treatments. A strength of the current study is the patient group, which allowed for a bias reduction due to the inclusion criteria. Our findings indicate that the provision and use of physiotherapy can be an important indicator of effective rehabilitation. However, the effect of rehabilitation can depend on experience and familiarity with neurofunctional exercises, such as PNF or Bobath. More research is required to determine the parameters that should be measured routinely to guide rehabilitation management in order to improve a patient’s quality of life after a stroke. This study’s limitations include the gender disparity seen between groups; the lack of blinding to the study design, which may increase the risk of performance and assessment bias; and the lack of functional outcome measures. Moreover, spasticity was assessed by only one experienced physiotherapist. This approach improves consistency but introduces potential observer bias, as there is no inter-rater assessment of reliability.

## 5. Conclusions

Our results endorse the use neurophysiological methods within standard rehabilitation frameworks as a highly effective approach to managing spasticity in post-stroke patients. They also support the opinion that integrating neurophysiological methods into the standard physiotherapy treatment of spasticity would be the most effective approach.

## Data Availability

The data can be provided upon a reasonable request to the corresponding author.
